# Water depth affects submersed macrophyte more than herbivorous snail in mesotrophic lakes

**DOI:** 10.3389/fpls.2024.1375898

**Published:** 2024-05-17

**Authors:** Wenjing Ren, Yiqian Yao, Xiaoyu Gao, Hao Wang, Zihao Wen, Leyi Ni, Xiaolin Zhang, Te Cao, Qingchuan Chou

**Affiliations:** ^1^ State Key Laboratory of Marine Resource Utilization in South China Sea, Hainan University, Haikou, China; ^2^ State Key Laboratory of Freshwater Ecology and Biotechnology, Institute of Hydrobiology, Chinese Academy of Sciences, Wuhan, China; ^3^ University of Chinese Academy of Sciences, Beijing, China

**Keywords:** submersed macrophytes, water depth, herbivory, periphyton, snail abundance

## Abstract

**Introduction:**

Water depth (WD) and snail abundance (SA) are two key factors affecting the growth of submersed aquatic plants in freshwater lake ecosystems. Changes in WD and SA drive changes in nutrients and other primary producers that may have direct or indirect effects on submersed plant growth, but which factor dominates the impact of both on aquatic plants has not been fully studied.

**Methods:**

To investigate the dominant factors that influence aquatic plant growth in plateau lakes, a one-year field study was conducted to study the growth of three dominant submersed macrophyte (i.e., *Vallisneria natans*, *Potamogeton maackianus*, and *Potamogeton lucens*) in Erhai Lake.

**Results:**

The results show that, the biomass of the three dominant plants, *P.maackianus*, is the highest, followed by *P.lucens*, and *V.natans* is the lowest. Meanwhile, periphyton and snails attached to *P.maackianus* are also the highest. Furthermore, WD had a positive effect on the biomass of two submersed macrophyte species of canopy-type *P.maackianus* and *P.lucens*, while it had a negative effect on rosette-type *V.natans.* Snail directly inhibited periphyton attached on *V.natans* and thereby increasing the biomass of aquatic plants, but the effect of snails on the biomass of the other two aquatic plants is not through inhibition of periphyton attached to their plants.

**Discussion:**

The dominant factors affecting the biomass of submersed macrophyte in Erhai Lake were determined, as well as the direct and indirect mechanisms of WD and snails on the biomass of dominant submersed macrophyte. Understanding the mechanisms that dominate aquatic plant change will have implications for lake management and restoration.

## Introduction

Submerged macrophytes serve as major primary producers in freshwater lakes and provide a range of ecological services and functions (Moss, 1990; Scheffer, 1998). For example, submerged macrophytes can provide extensive substrate, habitat, shelter and food resources for periphyton, invertebrates and fish, remove nutrients from the water column and sediment, inhibit sediment resuspension and improve water transparency, thereby influencing a series of ecological processes (Moss, 1990; Scheffer et al., 1993; Jeppesen et al., 1998). However, as with other components of freshwater ecosystems, submersed macrophyte growth limitation and biomass reduction can be influenced by a combination of biotic and abiotic factors, including WD, light, nutrients and macroinvertebrates (Zhang et al., 2020a; Yuan et al., 2021; Ren et al., 2022; Wen et al., 2022).

The distribution and growth of submersed macrophytes are closely linked to WD. WD affects submerged macrophytes both directly and indirectly by altering a number of other environmental variables, such as the intensity of underwater light and nutrients (Wang et al., 2019; Wang et al., 2021b; Zhang et al., 2022). Many studies have shown that deep water can attenuate underwater light intensity and inhibit the growth and spread of submersed macrophytes (Yuan et al., 2021; Chen et al., 2023). In addition, shallow areas of lakes are susceptible to wind and wave disturbance, which can cause changes such as sediment resuspension, reduced water transparency, and increased concentrations of nitrogen, phosphorus (Van Zuidam and Peeters, 2015; Xu et al., 2022; Zhou et al., 2022). Thus, differences in WD can affect the composition and biomass of submersed macrophytes in freshwater lakes by altering a variety of factors in the water column. Furthermore, the response and adaptability of submersed macrophytes with different growth forms to WD differ in freshwater ecosystems (He et al., 2019; Meng et al., 2023). *Vallisneria natans*, *Potamogeton maackianus* and *Potamogeton lucens* are three common and dominant submersed macrophytes in Erhai lake, Yunnan Province, China, and their growth forms are different: *V.natans* is a rosette-forming species, and *P.maackianus*, *P.lucens* shows canopy growth (Wang et al., 2021a; Yu et al., 2022; Wen et al., 2022). Therefore, there may be differences in the pathways affected by submersed macrophytes of different growth forms.

In addition to WD effects, variation in SA may also contribute to changes in aquatic macrophyte biomass (Sheldon, 1987; Zhi et al., 2020). In general, as common benthic invertebrates in freshwater lakes, snails are generally regarded as generalist herbivores with diverse food sources including organic detritus, periphyton, decaying and living aquatic macrophytes (Brönmark, 1990; Yang et al., 2020; Ren et al., 2022). A complex relationship among snails, periphyton and macrophytes has been shown in many previous studies (Li H. et al., 2020; Yang et al., 2020; Zhi et al., 2020; Ren et al., 2022). For example, snails can indirectly promote the growth of submersed macrophytes by directly scraping periphyton attached to the surface of aquatic plants (Yang et al., 2020; Ren et al., 2022). In addition, snails can also directly graze macrophytes thereby inhibiting aquatic plant growth, especially at high snail densities (Zhi et al., 2020). Furthermore, snails can increase nutrient levels in the water column through excretion, which is likely to affect the relationship between periphyton and macrophytes. However, early studies were relatively short, or only conducted only under controlled experimental conditions in mesocosms (Yang et al., 2020; Ren et al., 2022), and these studies do not necessarily reflect the interactions between snails, periphyton and macrophytes observed in filed lakes. Furthermore, different plants typically have different leaf complexity (Dibble and Thomaz, 2009), with increased periphyton biomass associated with higher leaf complexity in macrophytes (Ferreiro et al., 2013; Hao et al., 2017; Zhi et al., 2020). Thus, heterogeneity of macrophytes with different structures may influence the relationship among snail, periphyton, and macrophyte.

A large number of studies have been conducted on the effects of environmental factors such as water depth, light availability, snail abundance and water column nutrient content on the biomass, community structure and distribution of submersed aquatic plants (Olsen et al., 2015; Li et al., 2020a; Qin et al., 2020; Zhang et al., 2020b), and they have primarily focused on a single factor or in the mesocosm experiment (Yu et al., 2015; Zhi et al., 2020; Zhang et al., 2020b; Ren et al., 2022; Chen et al., 2023). However, the comprehensive effects of WD, SA and other environmental factors on submersed aquatic plants are still unknown in plateau lakes with complex ecological processes and require investigation in field lakes.

Erhai is a large plateau lake with important ecological significance in Yunnan Province, China (Li et al., 2020b). The declines of submersed macrophytes due to anthropogenic activities and eutrophication has been repeatedly reported (Fu et al., 2018). In our field survey, *P.maackianus*, *P.lucens*, and *V.natans* were found to be the top three dominant submersed macrophytes in Erhai lake. Therefore, we conducted a one-year quarterly survey and measured the biomass of the three aquatic plants species in different WD, as well as periphyton, snail and the other environmental factors. In the present study, we identify the dominant environmental factors that influence the respective biomass of different growth forms in freshwater lakes. we hypothesized that 1) The effects of WD on submersed macrophyte biomass vary among species; 2) Snails promote submersed macrophyte growth by grazing on periphyton, but different species of aquatic plant species may be affected differently; 3) WD may have a dominant effect on the biomass of submersed plants with different growth forms rather than SA, because the distribution of snails may be affected by water depth (according to our field observations).

## Materials and methods

### Study area

**Figure 1:**
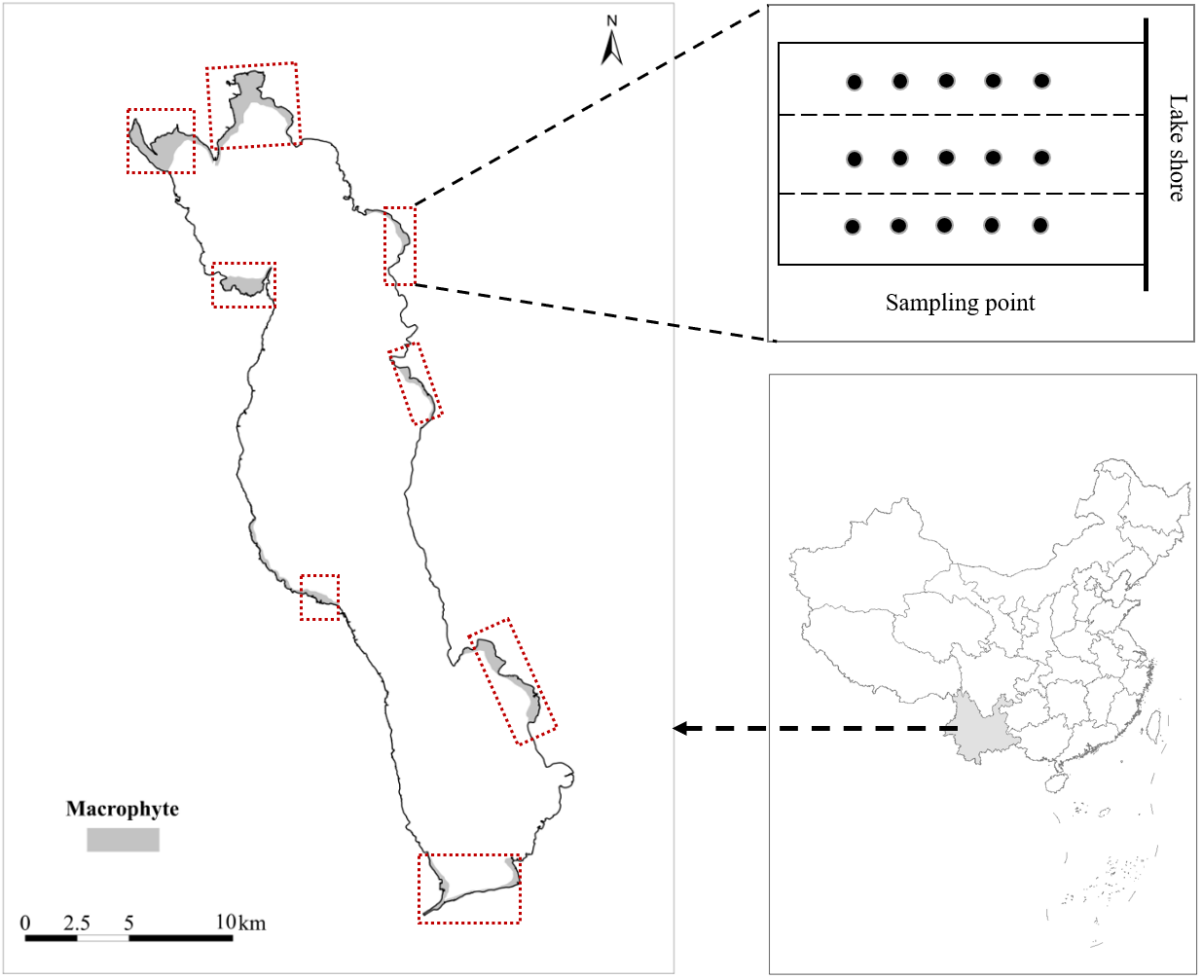
The location of Erhai Lake and sampling bays.

The study was conducted out at Erhai Lake, Yunnan Province, China (25°55′N,100°78′E). Erhai Lake is a large subtropical freshwater lake in China. It covers a water surface area of 252 km^2^ and has an average depth of 10 m (with a maximum depth of 20 m). In recent decades, external nutrient inputs to the lake have also increased as a result of increased anthropogenic activities, and the water quality of Erhai Lake has changed from oligotrophic to mesotrophic (Lin et al., 2020), which caused the submersed macrophyte community to shift from being dominated by *Potamogeton maackianus* to being jointly dominated by *V.natans* and *P.maackianus* (He et al., 2015; Wang et al., 2021a). From September 2019 to August 2020, we conducted quarterly surveys and sampling in eight bays around Erhai Lake for one year to investigate the effects of water depth and snail abundance on the three dominant plants in Erhai Lake. The location of Erhai Lake, the distribution of sampling bays and sampling points are shown in **Figure 1**.

### Measurement of water physiochemical parameters and phytoplankton biomass

We designed three parallel transects in each bay, and the sampling points on each transect were set at intervals of 1m WD. The sampling WD are 1 m, 2 m, 3 m, 4 m, 5 m, and 6 m respectively. Prior to water sampling, WD was measured at each site and 2 L of mixed water samples were collected at 50 cm below the water surface, at the intermediate depth and at 50 cm above the sediment surface. The collected water samples were acidified and stored at 4°C, and transported to the laboratory as soon as possible, and the water column concentrations of total nitrogen (TN), total phosphorus (TP), orthophosphate (PO_4_-P), nitrate (NO_3_-N) and ammonium nitrogen (NH_4_-N) were determined in accordance with standard methods. After filtering the water samples through Whatman GF/C glass fiber filters, chlorophyll-a (Chl a) was determined by the ethanol-thermal method (Liu et al., 2020).

Use a water quality analyzer (YSI, USA) to measure physical and chemical indicators of the water column including pH, dissolved oxygen (DO), conductivity (C), total dissolved solids (TDS) and oxidation-reduction potential (ORP) at each sampling point in the field. Photosynthetically active radiation (PAR) was determined at 0.5 m WD intervals (0, 0.5, 1, 1.5, 2, 2.5 … m) on the basis of the actual WD of the sampling point with the Li-1400 data logger (Li-1400; Li-Cor Company, Lincoln, NE, USA). The calculation equation for the light extinction coefficient (K) of the water column is as follows: K = -(1/d) ln (I_d_/I_s_). In the equation, d is the water depth, I_d_ is the PAR value at the water depth d, and Is is the PAR value at the water surface (Krause-Jensen et al., 1998).

### Measurements of macrophytes, periphyton and snails

Submersed macrophytes were collected with a rotating harvester hook (sampling area: 0.2 m^2^) at each sampling site after water sampling was completed. The plants obtained were cleaned and identified according to species, and then the three dominant species (*V.natans, P.maackianuss* and *P.lucens*) were selected and weighed separately, and then placed in sealed bags and brought to the laboratory for subsequent processing. Furthermore, prior to bagging, one plant per variety was chosen at random, individually placed in a sealed plastic bag and brought to the laboratory, and then tap water was added to the sealed bag several times to remove periphyton attached to the plant by shaking thoroughly. The periphyton solution obtained after several shakes was fixed with Lugol’s reagent, precipitated for 48 hours and concentrated to a final volume of 40-50 ml for storage. The mass and abundance of the periphyton was then calculated by counting the concentrated periphyton solution using a counting chamber (0.1 ml) under a microscope at 400 times magnification. Periphyton biomass was calculated as the mass of periphyton attached to the plants divided by the weight of the host plant and expressed in mg g^−1^. Finally, three species of the plants, which were packed separately and returned to the laboratory, were further processed to collect the snails. Each species of submersed macrophytes brought back to the laboratory in sealed bags were rinsed several times, and the rinsed solution was filtered through a sieve with a pore size of 1 mm. At the end, the snails collected in the sieve were counted and weighed.

### Data analysis

Homogeneity of variance and normal distribution tests were conducted on all data before analysis. Before conducting variance analysis and constructing structural equations, the data of three aquatic plants, snail abundance, periphyton biomass, and water nutrients were all log (x+1) transformed to meet the data analysis requirements. Differences in the biomass of the three submersed macrophytes *V.natans, P.maackianuss* and *P.lucens*, as well as periphyton and snail attachment to the three plants and environmental variables (TN, TP, Chl a, and K), were analyzed using one-way ANOVA and non-parametric tests. A Kruskal-Wallis test was performed using the “ggpubr” package to determine differences in in biomass and their periphyton and snail among the three species and environmental variables.

The R package “lmerTest” was used for the following analysis. A multivariate linear mixed model was constructed to detect the effects of WD, snail, nutrients (TN and TP), and light (K) on the biomass of three macrophyte specie and their periphyton biomass, using as a random variable. The standardized regression coefficient for each explanatory variable implies the change in the average response per unit increase in the associated predictor variable, holding all other predictor variables constant. In addition, we quantified the inclusive R2 for WD, snail, nutrients (TN and TP) and light (K) based on linear mixed effects model fitting using the “partR2” package in R. The inclusive R2 here is the variance explained by the predictor without taking into account covariance with other predictors. Higher values of inclusive R2 indicate that a predictor is more important in explaining the variance of macrophyte biomass (*V.natans, P. maackianuss* and *P.lucens*).

Structural equation modeling (SEM) was used to analyze the effects of WD, nutrients and snails on the biomass of three submersed aquatic plants in subtropical lakes. We used the R package “piecewiseSEM” to construct the SEM, adding a random effect of season in each path. An overall test of fit of the SEM was performed using Fisher’s c, degrees of freedom, and p-values.

## Results

### Difference in biomass of *V.natans*, *P.maackianuss*, and *P.lucens*

**Figure 2:**
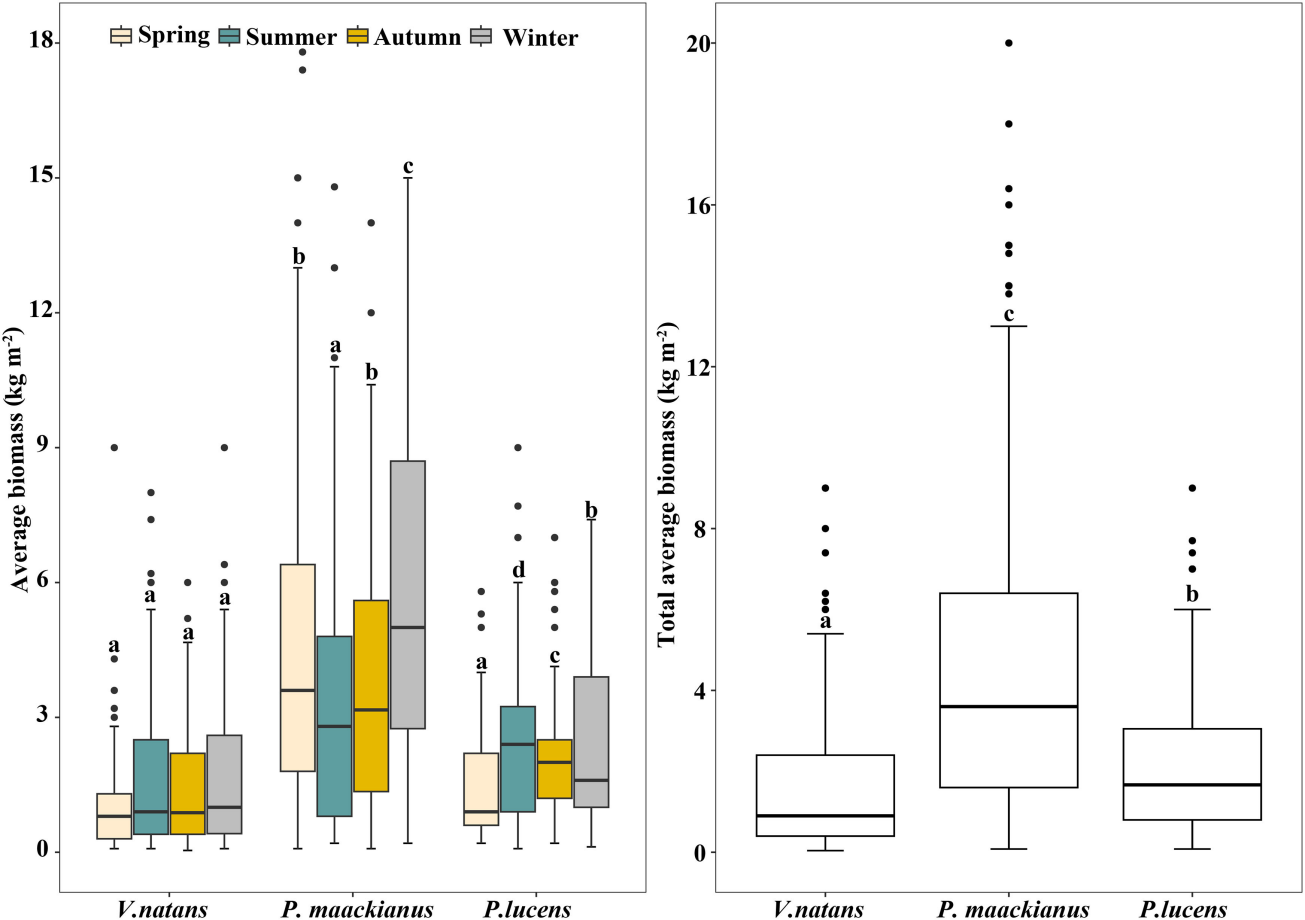
Biomass of three dominant submersed macrophytes in Erhai Lake across four seasons. Mean and standard deviation are shown in each bar. The different letters on the bars indicate significant differences at p < 0.05.

The biomass of the three submersed macrophyte species showed different variation trends in the four seasons (**Figure 2**). The biomass of *V.natans* has no significant difference among four seasons, with an average biomass of 1.64kg m^-2^. Meanwhile, there were significant differences in the biomass of *P.maackianuss* and *P.lucens* among the four seasons. Among them, the biomass of *P.maackianuss* was highest in spring and winter, and lowest in summer and autumn, with values of 4.89-5.86 kg m^-2^ and 3.49-3.91kg m^-2^ respectively, whereas the biomass of *P.lucens* was opposite, with biomass of 1.2-1.59 kg m^-2^ in spring and winter and 2.30-2.50 kg m^-2^ in summer and autumn. Over the four seasons, the average of *V.natans*, *P.maackianuss* and *P.lucens* biomass was 1.64 ± 1.58 kg m^-2^, 4.62 ± 3.91 kg m^-2^ and 2.32 ± 1.83 kg m^-2^, respectively.

### Difference in snail and periphyton attached on *V.natans*, *P.maackianuss*, and *P.lucens*

**Figure 3:**
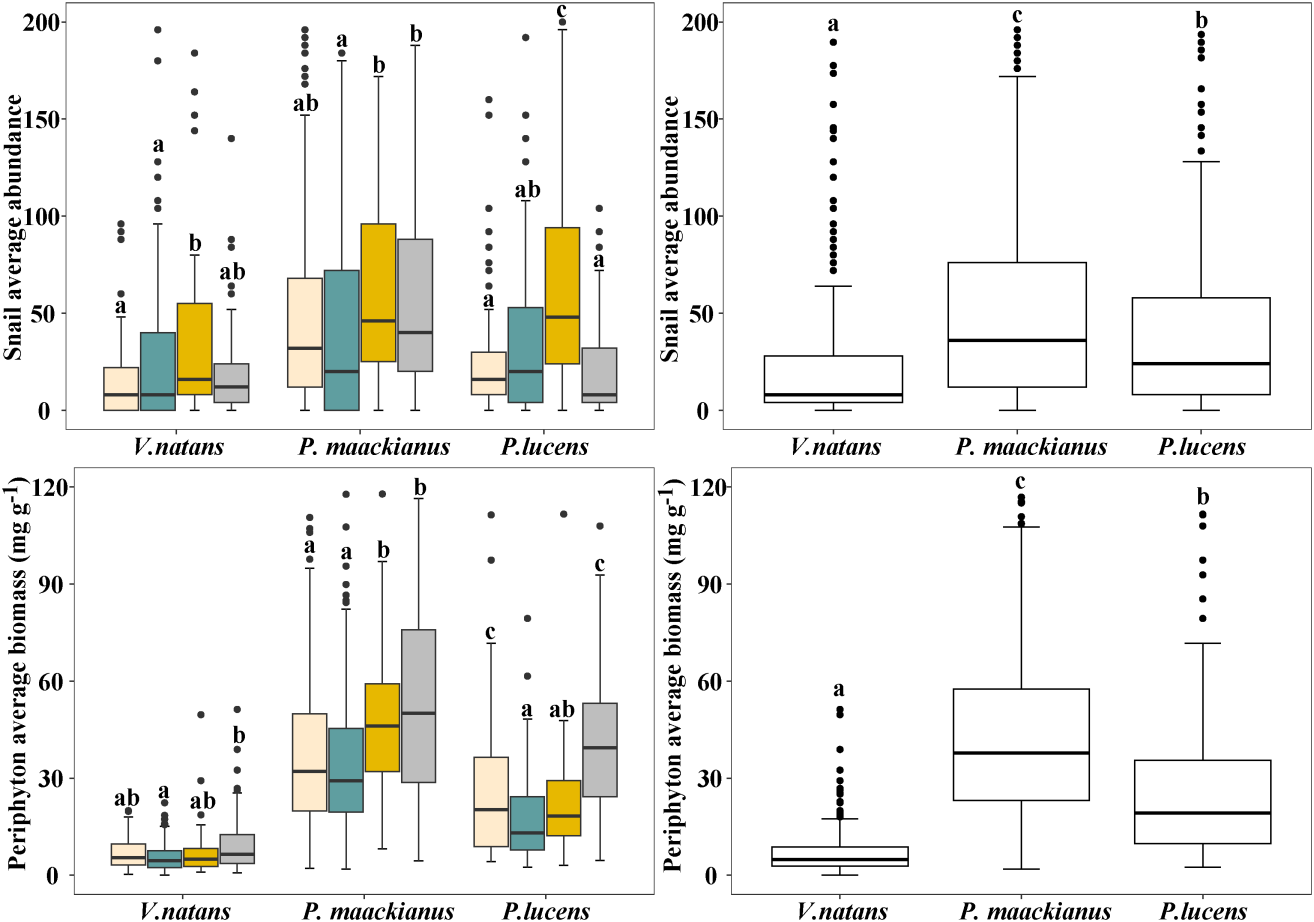
Changes in SA and periphyton biomass attached to three submerged macrophytes. Mean and standard deviation are shown in each bar. The different letters on the bars indicate significant differences at p < 0.05.

There were significant differences in SA and periphyton biomass on the three species among the four seasons (**Figure 3**) . Snail abundance and periphyton biomass on three submerged macrophyte species (with similar trends) were higher in autumn and winter than in spring and summer, and no major differences were found between spring summer and autumn winter, except for *P.lucens*. The abundance of snails on *P.lucens* was significantly lower in winter than in autumn, while the opposite was true for periphyton biomass. In general, there were significant differences in the average SA and periphyton biomass on the three submersed macrophyte species. The average SA and periphyton biomass on *V.natans* was the lowest, 7.04 mg g^-1^, followed by *P.lucens*, and the highest was *P.maackianus.*

### Changes in environmental factors

**Figure 4:**
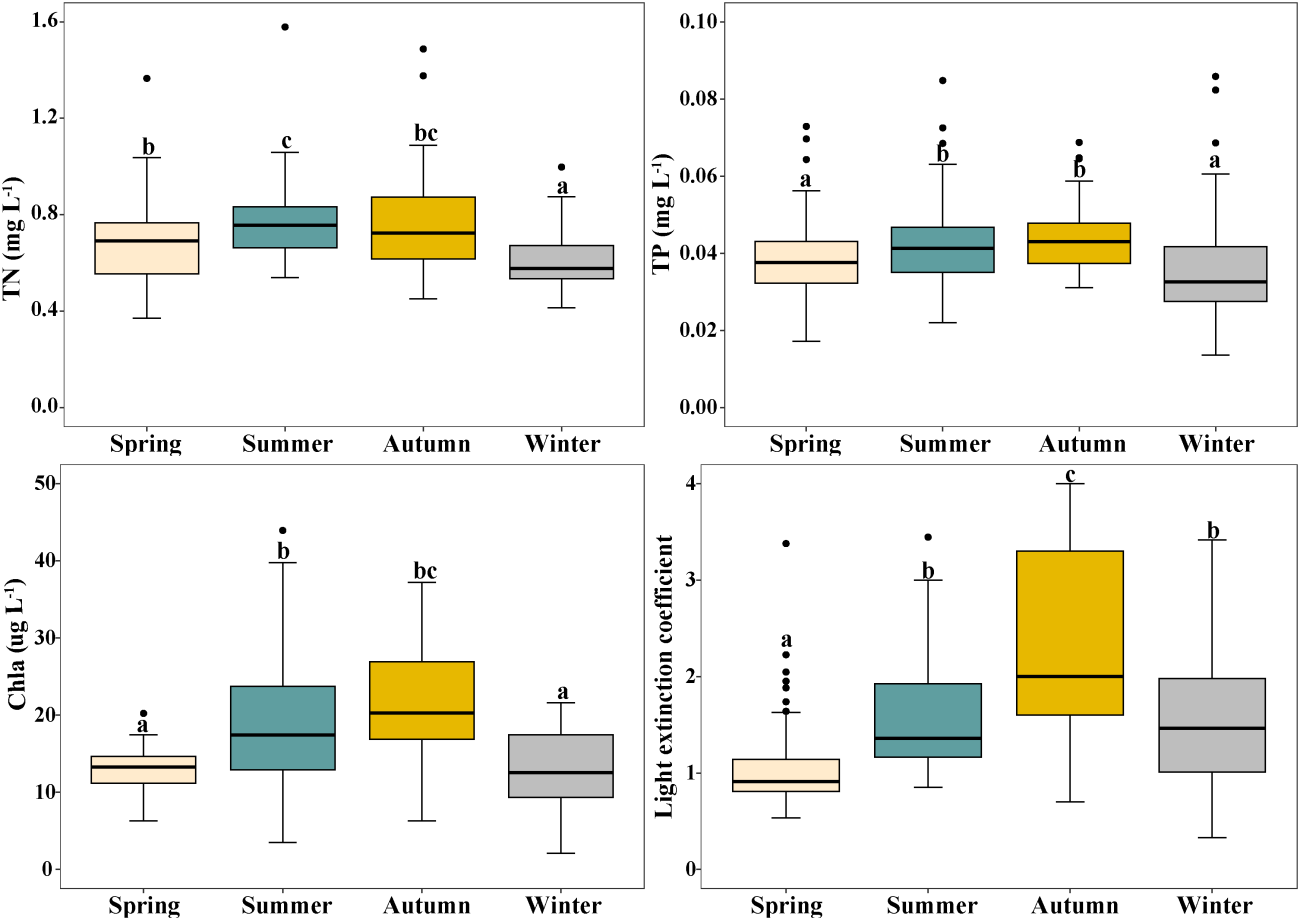
Changes in total nitrogen (TN), total phosphorus (TP), phytoplankton chlorophyll- a (Chl a) and light extinction coefficient (K) in Erhai Lake across four seasons. Mean and standard deviation are shown in each bar. The different letters on the bars indicate significant differences at p < 0.05.

Overall, TN, TP, Chl a and light extinction coefficient (K) differed significantly among the four seasons, and showed similar trends of variation, with the highest values in summer and autumn and lowest values in spring and winter. For TN, 0.76 mg L^−1^ and 0.63 mg L^−1^ were the highest and lowest values, respectively. The highest and lowest values for TP were 0.043 mg L^−1^ and 0.035 mg L^−1^, respectively (**Figure 4**). The highest values of Chl a and K were 20.3 mg L^-1^ and 2.15, respectively, and the lowest values were 12.7 mg L^-1^ and 0.95 (**Figure 4**).

### Effects of multiple driving factors on macrophytes biomass

**Figure 5:**
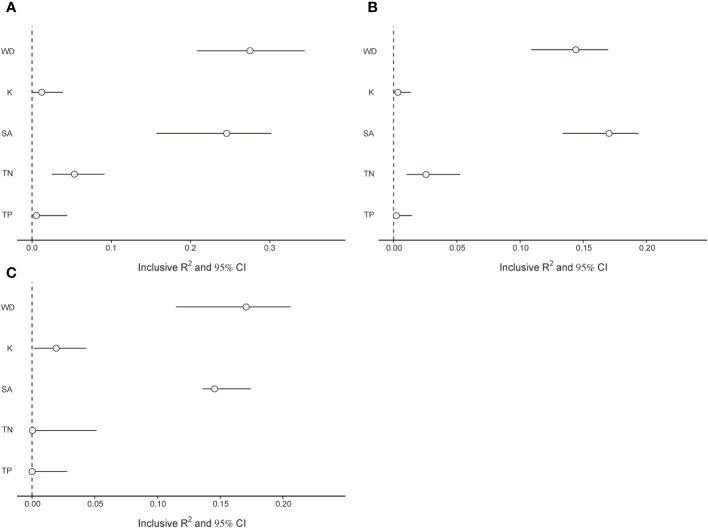
Inclusive R2 and 95% CI for each driver on **(A)** *V.natans* biomass and **(B)** *P.maackianuss* biomass and **(C)** *P.lucens* biomass.

The multivariate linear mixed model showed that WD had a significant negative effect on *V.natans* biomass (β= -0.22, p<0.01), while it had a significant positive effect on *P.maackianus* (β= 0.124, p<0.001) and *P.lucens* biomass (β= 0.182, p<0.05) (**Table 1**). Meanwhile, WD had a significant negative effect on all periphyton (β= -0.21, p<0.001; β= -0.135, p<0.001), except for periphyton attached on *P.lucens* (β= -0.067, p>0.05). SA had a significant positive effect on the biomass of three plants (*V.natans*: β= 0.28, p<0.01; *P.maackianus*: β= 0.329, p<0.001; *P.lucens*: β= 0.368, p<0.001) and periphyton attaches to *P.maackianus* (β= 0.143, p<0.001). For nutrients, TP has a positive effect on periphyton attached to the three plant species (β = 0.116, p < 0.05; β = 0.09, p < 0.05; β = 0.124, p < 0.05) and phytoplankton (Chl a: β = 0.236, p < 0.001) (**Table 1**). Among the five variables, we found that WD and SA were the most important drivers affecting three plant species, accounting for 14.8-27.2%, 14.7-24.4% of the variance respectively (**Figure 5**).

**Table 1:** Effect of water depth (WD), snail abundance (SA), nutrients (TN and TP), and light extinction coefficient (K) on three species macrophytes biomass and their periphyton biomass in a multivariate linear mixed model.

	n	R2 of fixed effects	Intercept	WD	K	SA	TN	TP
*V.natans* biomass	328	0.358	-0.015	-0.22^**^	0.076	0.28^**^	0.2^*^	0.2^*^
Periphyton on *V.natans*	328	0.256	1.25^***^	-0.21^***^	0.011	0.052	0.069	0.116^*^
*P.maackianus* biomass	576	0.52	-0.007	0.124^*^	0.042	0.329^***^	-0.11	-0.072
Periphyton on *P.maackianus*	576	0.144	1.78^***^	-0.135^**^	-0.058	0.143^***^	-0.028	0.09^*^
*P.lucens* biomass	184	0.517	-0.169	0.182^*^	0.096	0.368^***^	0.077	-0.011
Periphyton on *P.lucens*	184	0.654	1.544^***^	-0.067	-0.255^**^	-0.028	0.423^*^	0.124^*^
Chl a	312	0.457	0.949^***^	0.34^***^	-0.151^*^	-0.04	0.056	0.236^***^

Data were standardized before analysis. Significance level: *** = p < 0.001; ** = p < 0.01; * = p < 0.05.

### Effect of snail, nutrients and water depth on macrophytes biomass

**Figure 6:**
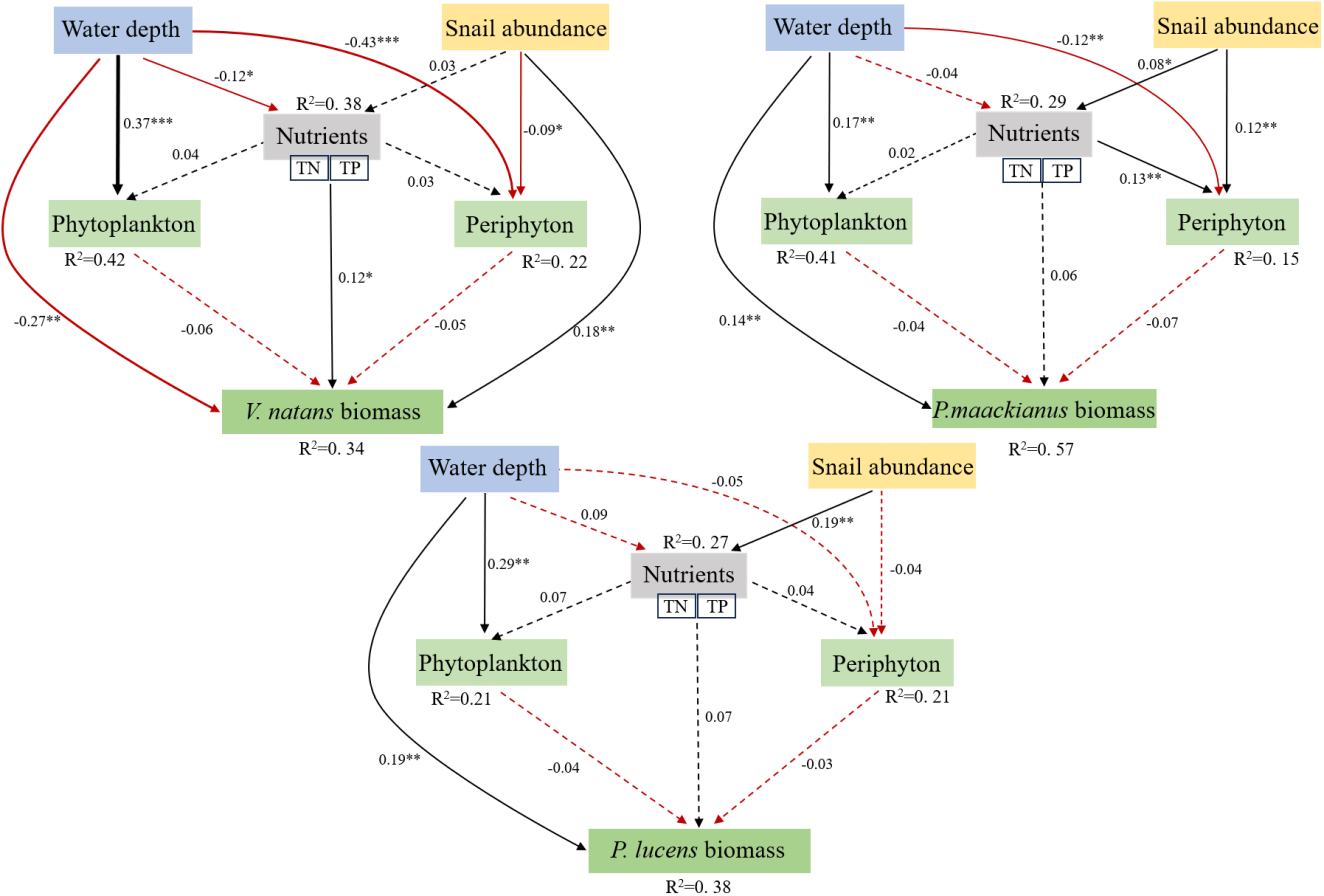
Piecewise structural equation model (SEM) showing the effects of WD, SA and nutrients on macrophyte biomass directly and indirectly. Solid black line shows a significant positive correlation and the solid red line shows a significant negative correlation, with the dotted line indicates no significance. Standardized path coefficients (similar to relative regression weights) are shown as numbers next to the path lines. Next to each response variable in the model is the proportion of variance explained (R2). Model fit: *V.natans*: Fisher’s C = 2.139, P=0.508; *P. maackianus*: Fisher’s C = 0.469, P=0.791; *P.lucens*: Fisher’s C = 0.214, P=0.899. *p < 0.0 5, **p < 0.01, ***p < 0.001.

Piecewise SEM results showed that WD and SA could have direct or indirect effects on macrophyte biomass (**Figure 6**). Overall, the model explained 34%, 57%, and 38% of the variation in biomass of the three aquatic species, respectively. Specifically, WD had a direct negative effect on *V.natans*, but a significant positive effect on *P.maackianus* and *P.lucens*. SA directly inhibits periphyton attached to *V.natans* and thereby increasing the biomass of aquatic plants, but the effect of snails on the biomass of the other two aquatic plants is not through inhibition of periphyton attached to their plants.

## Discussion

Our results showed that WD inhibited the growth of *V.natans*, but significantly increased the biomass of *P.maackianus* and *P.lucens*. SA significantly inhibited periphyton attached to *V.natans*, thereby enhancing its host plant biomass, but it was difficult to offset the negative effect of WD on *V.natans*. Meanwhile, snails did not have a positive impact on their hosts by significantly inhibiting the periphyton attached to the *P.maackianus* and *P.lucens.* Overall, the three dominant species are mainly affected by WD, which means that WD has a greater impact on submersed plants than snail grazing in Erhai Lake, a mesotrophic lake.

WD is one of the most important factors influencing the growth and distribution of submersed macrophytes (Olsen et al., 2015; Su et al., 2018; Li et al., 2020a; Qin et al., 2020), and many studies has shown that variation of WD can have both an effect on underwater light availability and play an important role in the nutrient status of lakes (Li et al., 2020a; Qin et al., 2020; Yang et al., 2022; Chen et al., 2023). Generally, with the increase of water depth, the intensity of underwater light decreases, which is not conducive to the growth of submersed macrophytes (Yuan et al., 2021; Chen et al., 2023). In our results, WD significantly inhibited the biomass of *V.natans* biomass while at the same time significantly improving the biomass of *P.maackianus* and *P.lucens*. The reason for the inconsistent results on the effect of water depth on different plant biomass may be that the different growth types of the dominant macrophytes respond differently to underwater light caused by WD. *V.natans*, as a rosette-type submersed macrophyte with its stems and leaves under water, responds to changes in water depth by regulating leaves chlorophyll a concentration and photosynthetic efficiency, whereas *P.maackianus* and *P.lucens*, as canopy-type submersed plants, respond to changes in water depth by extending their shoot length to the water surface (Chen et al., 2016; He et al., 2019). In addition, *V.natans* biomass was the lowest among the three dominant macrophytes, followed by *P.lucens*, and *P.maackianus* biomass was the highest. This indicates that within the range of 0-6m water depth, the attenuation of underwater light intensity caused by deeper WD has a greater inhibitory effect on rosette-type plants than on canopy-type plants. In other words, rosette-type *V.natans* is more susceptible to deeper WD and light suppression. For example, Li et al. (2021) found strong suppression of both *V.natans* and *H.verticillata* biomass in deeper water (250cm), whereas only *V.natans* was suppressed in intermediate water depth (150cm). Moreover, we also found nutrient significantly enhanced the growth of *V.natans* and had no significant effect on the other two plants, suggesting that *V.natans* may have a broad nutrient-absorption capacity. The adaptation of these species to their environment may explain the differences in biomass.

The impact of snails on aquatic plants depends on the snail abundance (Jones and Sayer, 2003; Zhi et al., 2020). While macrophytes can also be used as a food source by snails, it is generally accepted that periphyton constitutes a larger proportion of the snail diet than macrophytes. For example, Zhi et al. (2020) reported an effective removal of periphyton from four macrophyte species (*Myriophyllum spicatum, Potamogeton wrightii, P. crispus*, and *P. oxyphyllus*) by herbivorous snails, resulting in a significant increase in host plant biomass. Nevertheless, high densities of snails can consume both plants and periphyton, resulting in no positive effect on submerged macrophytes (Zhi et al., 2020). In this study, we observed that when the abundance of snails attached to *V.natans* is approximately 13 ind m^-2^, they exerted significant feeding pressure on the periphyton, and contributed to an increase in host plant biomass. This is also in line with the results of previous studies. For example, Ren et al. (2022) found when the initial density of herbivorous snail was 16 ind m^-2^, *V.natans* biomass could be promoted by removing periphyton attached to host plant leaves. However, despite the higher abundance of snails on *P.maackianus* (45 ind m^-2^) and *P.lucens* (32 ind m^-2^), there was no obvious effect on the periphyton and their host plants. This may also be due to the fact that snail have other abundant food sources besides periphyton and their host plants, which may require further verification.

Changes in WD are often accompanied by changes in the physical and chemical parameters of the water column, which will inevitably affect the growth of submersed macrophyte, either directly or indirectly (Meng et al., 2023). For example, external disturbances such as wind and waves tend to resuspend sediments and release nutrients (TN and TP) from the sediments into the water column, reducing clarity in shallow areas (Tammeorg et al., 2015; Baastrup Spohr et al., 2016; Tong et al., 2017). Our results found that the concentration of TN, TP and Chla showed similar trends of variation, with the highest values in summer and autumn and lowest values in spring and winter. In addition, the average concentrations of TN and TP (0.76 mg L^-1^ and 0.036 mg L^-1^, respectively) were highest in the depth range 0-2m, which were higher than those in the depth range 2-4m and 4-6m. Meanwhile, *V.natans* biomass was the highest in 0-2m water depth (consistent with the change of TN and TP), while the biomass of *P.maackianus* and *P.lucens* were the highest in 2-4m and 4-6m, respectively. Although the concentration of TN and TP in the water column strongly influence the biomass of *V.natans*, and snails also promote *V.natans* growth by inhibiting periphyton, but it is difficult to offset the direct negative effects of WD on *V.natans*. In addition, we found that WD significantly increased the biomass of *P.maackianus* and *P.lucens*, while SA significantly affected the nutrient contents in the water column but did not significantly affect *P.maackianus* and *P.lucens*, indicating that aquatic plants are directly controlled by WD and less affected by nutrient contents and SA.

In conclusion, the three dominant aquatic plants in a meso-eutrophic lake Erhai were mainly and directly affected by WD, and snail only had a positive and direct promotion effect on the rosette-type *V.natans* by grazing the periphyton attached to its host plant, but had no significant effect on the other two canopy-type submersed plants (*P.maackianus* and *P.lucens*). WD can directly inhibit the increase of *V.natans* biomass, but significantly increased the biomass of *P.maackianus* and *P.lucens*. SA significantly inhibited periphyton attached to *V.natans*, thereby increasing aquatic plant biomass, but it was difficult to offset the negative effect of WD on *V.natans*. For *P.maackianus* and *P.lucens*, although SA significantly affected the nutrient content of the water column, it did not significantly affect the biomass of *P.maackianus* and *P.lucens*. Overall, aquatic plants are directly controlled by WD and less affected by nutrient contents and SA in the mesotrophic lake.

## Data availability statement

The raw data supporting the conclusions of this article will be made available by the authors, without undue reservation.

## Author contributions

WR: Investigation, Methodology, Writing – original draft, Writing – review & editing, Conceptualization, Data curation, Formal analysis, Funding acquisition, Project administration, Resources, Software, Supervision, Validation, Visualization. YY: Investigation, Methodology, Writing – original draft, Writing – review & editing. XG: Methodology, Investigation, Writing – original draft, Writing – review & editing. HW: Formal analysis, Methodology, Writing – original draft, Writing – review & editing. ZW: Formal analysis, Methodology, Writing – original draft, Writing – review & editing. LN: Supervision, Writing – original draft, Writing – review & editing. XZ: Supervision, Writing – original draft, Writing – review & editing. TC: Supervision, Writing – original draft, Writing – review & editing. QC: Supervision, Writing – original draft, Writing – review & editing.

## References

[B1] Baastrup Spohr L. Moller C. L. Sand Jensen K. (2016). Water-level fluctuations affect sediment properties, carbon flux and growth of the isoetid Littorella uniflora in oligotrophic lakes. Freshw. Biol. 61, 301–315. doi: 10.1111/fwb.12704

[B2] Brönmark C. (1990). How do herbivorous freshwater snails affect macrophytes?–A comment. Ecology 71, 1212–1215. doi: 10.1111/fwb.12704

[B3] Chen J. Cao T. Zhang X. Xi Y. Ni L. Jeppesen E. (2016). Differential photosynthetic and morphological adaptations to low light affect depth distribution of two submersed macrophytes in lakes. Sci. Rep. 6, 34028. doi: 10.1038/srep34028 27694880 PMC5046178

[B4] Chen S. Jiang L. Ma S. Wu Y. Ye Q. Chang Y.. (2023). Response of a submersed macrophyte (Vallisneria natans) to water depth gradients and sediment nutrient concentrations. Sci. Total Environ. 912, 169154–169154. doi: 10.1016/j.scitotenv.2023.169154 38065501

[B5] Dibble E. D. Thomaz S. M. (2009). Use of fractal dimension to assess habitat complexity and its influence on dominant invertebrates inhabiting tropical and temperate macrophytes. J. Freshw. Ecol. 24, 93–102. doi: 10.1080/02705060.2009.9664269

[B6] Ferreiro N. Giorgi A. Feijoo C. (2013). Effects of macrophyte architecture and leaf shape complexity on structural parameters of the epiphytic algal community in a Pampean stream. Aquat. Ecol. 47, 389–401. doi: 10.1007/s10452-013-9452-1

[B7] Fu H. Yuan G. Lou Q. Dai T. Xu J. Cao T.. (2018). Functional traits mediated cascading effects of water depth and light availability on temporal stability of a macrophyte species. Ecol. Indic. 89, 168–174. doi: 10.1016/j.ecolind.2018.02.010

[B8] Hao B. Wu H. Cao Y. Xing W. Jeppesen E. Li W. (2017). Comparison of periphyton communities on natural and artificial macrophytes with contrasting morphological structures. Freshw. Biol. 62, 1783–1793. doi: 10.1111/fwb.12991

[B9] He L. Zhu T. Cao T. Li W. Zhang M. Zhang X.. (2015). Characteristics of early eutrophication encoded in submersed vegetation beyond water quality: a case study in Lake Erhai, China. Environ. Earth Sci. 74, 3701–3708. doi: 10.1007/s12665-015-4202-4

[B10] He L. Zhu T. Wu Y. Li W. Zhang H. Zhang X.. (2019). Littoral slope, water depth and alternative response strategies to light attenuation shape the distribution of submersed macrophytes in a mesotrophic lake. Front. Plant Sci. 10. doi: 10.3389/fpls.2019.00169 PMC6391712 30842784

[B11] Jeppesen E. Jensen J. P. Sndergaard M. Lauridsen T. Sandby K. (1998). Changes in nitrogen retention in shallow eutrophic lakes following a decline in density of cyprinids. Archiv fur Hydrobiol. 142, 129–151. doi: 10.1127/archiv-hydrobiol/142/1998/129

[B12] Jones J. I. Sayer C. D. (2003). Does the fish–invertebrate–periphyton cascade precipitate plant loss in shallow lakes? Ecology 84, 2155–2167. doi: 10.1890/02-0422

[B13] Krause-Jensen D. Sand-Jensen K. J. L. Oceanography (1998). Light attenuation and photosynthesis of aquatic plant communities. Limnol. Oceanogr. 43, 396–407. doi: 10.4319/lo.1998.43.3.0396

[B14] Li H. Li Q. Luo X. Fu J. Zhang J. (2020a). Responses of the submersed macrophyte Vallisneria natans to a water depth gradient. Sci. Total Environ. 701, 134944. doi: 10.1016/j.scitotenv.2019.134944 31715481

[B15] Li J. Bai Y. Alatalo J. M. (2020b). Impacts of rural tourism-driven land use change on ecosystems services provision in Erhai Lake Basin, China. Ecosyst. Serv. 42, 101081. doi: 10.1016/j.ecoser.2020.101081

[B16] Li Q. Han Y. Chen K. Huang X. Li K. He H. (2021). Effects of water depth on the growth of the submerged macrophytes vallisneria natans and hydrilla verticillata: implications for water level management. Water 13 (18), 2590. doi: 10.3390/w13182590

[B17] Lin S. Shen S. Zhou A. Lyu H. (2020). Sustainable development and environmental restoration in Lake Erhai, China. J. Clean. Prod. 258, 120758. doi: 10.1016/j.jclepro.2020.120758

[B18] Liu H. Zhou W. Li X. Chu Q. Tang N. Shu B.. (2020). How many submersed macrophyte species are needed to improve water clarity and quality in Yangtze floodplain lakes? Sci. Total Environ. 724, 138267. 32247982 10.1016/j.scitotenv.2020.138267

[B19] Meng Z. Yu X. Xia S. Zhang Q. Ma X. Yu D. (2023). Effects of water depth on the biomass of two dominant submersed macrophyte species in floodplain lakes during flood and dry seasons. Sci. Total Environ. 877, 162690–162690. doi: 10.1016/j.scitotenv.2023.162690 36894075

[B20] Moss B. (1990). Engineering and biological approaches to the restoration from eutrophication of shallow lakes in which aquatic plant communities are important components Biomanipulation Tool for Water Management. Hydrobiology 200, 367–377. doi: 10.1007/BF02530354

[B21] Olsen S. Chan F. Li W. Zhao S. Sondergaard M. Jeppesen E. (2015). Strong impact of nitrogen loading on submersed macrophytes and algae: a long-term mesocosm experiment in a shallow Chinese lake. Freshw. Biol. 60, 1525–1536. doi: 10.1111/fwb.12585

[B22] Qin B. Zhou J. Elser J. J. Gardner W. S. Deng J. Brookes J. D. (2020). Water depth underpins the relative roles and fates of nitrogen and phosphorus in lakes. Environ. Sci. Technol. 54, 3191–3198. doi: 10.1021/acs.est.9b05858 32073831

[B23] Ren W. Wen Z. Cao Y. Wang H. Yuan C. Zhang X.. (2022). Cascading effects of benthic fish impede reinstatement of clear water conditions in lakes: A mesocosm study. J. Environ. Manage. 301, 113898. doi: 10.1016/j.jenvman.2021.113898 34626943

[B24] Scheffer M. (1998). Ecology of Shallow Lakes (Dordrecht: Kluwer Academic Publishers).

[B25] Scheffer M. Hosper S. H. Meijer M. L. Moss B. Jeppesen E. (1993). Alternative equilibria in shallow lakes. Trends Ecol. Evol. 8, 275–279. doi: 10.1016/0169-5347(93)90254-M 21236168

[B26] Sheldon S. P. (1987). The effects of herbivorous snails on submersed macrophyte communities in Minnesota Lakes. Ecology 68, 1920–1931. doi: 10.2307/1939883 29357136

[B27] Su H. Zhu T. Bai X. Ni L. Xie P. Zhang X. (2018). Seed germination indicates adaptive transgenerational plasticity in a submersed macrophyte. Front. Plant Sci. 9, 1592. doi: 10.3389/fpls.2018.01592 30519247 PMC6258819

[B28] Tammeorg O. Horppila J. Laugaste R. Haldna M. Niemisto J. (2015). Importance of diffusion and resuspension for phosphorus cycling during the growing season in large, shallow Lake Peipsi. Hydrobiologia 760, 133–144. doi: 10.1007/s10750-015-2319-9

[B29] Tong Y. Liang T. Wang L. Li K. (2017). Simulation on phosphorus release characteristics of Poyang Lake sediments under variable water levels and velocities. J. Geogr. Sci. 27, 697–710. doi: 10.1007/s11442-017-1401-9

[B30] Van Zuidam B. G. Peeters E. T. H. M. (2015). Wave forces limit the establishment of submersed macrophytes in large shallow lakes. Limnol. Oceanogr. 60, 1536–1549. doi: 10.1002/lno.10115

[B31] Wang H. Fu H. Wen Z. H. Yuan C. B. Zhang X. L. Ni L. Y.. (2021a). Seasonal patterns of taxonomic and functional beta diversity in submerged macrophytes at a fine scale. Ecol. Evol. 11 (14), 9827–9836. doi: 10.1002/ece3.7811 34306665 PMC8293774

[B32] Wang L. Han Y. Yu H. Fan S. Liu C. (2019). Submersed vegetation and water quality degeneration from serious flooding in Liangzi Lake, China. Front. Plant Sci. 10, 1504. doi: 10.3389/fpls.2019.01504 31824535 PMC6886514

[B33] Wang S. Gao Y. Jia J. Kun S. Lyu S. Li Z.. (2021b). Water level as the key controlling regulator associated with nutrient and gross primary productivity changes in a large floodplain-lake system (Lake Poyang), China. J. Hydrol. 599, 126414. doi: 10.1016/j.jhydrol.2021.126414

[B34] Wen Z. Wang H. Zhang Z. Cao Y. Yao Y. Gao X.. (2022). Depth distribution of three submersed macrophytes under water level fluctuations in a large plateau lake. Aquat. Bot. 176, 103451. doi: 10.1016/j.aquabot.2021.103451

[B35] Xu L. Hu Q. Liao L. Duan Z. Liu S. Chen L.. (2022). Hydrological isolation affected the chemo-diversity of dissolved organic matter in a large river-connected lake (Poyang Lake, China). Sci. Total Environ. 851, 158047. doi: 10.1016/j.scitotenv.2022.158047 35985600

[B36] Yang L. He H. Guan B. Yu J. Yao Z. Zhen W.. (2020). Mesocosm experiment reveals a strong positive effect of snail presence on macrophyte growth, resulting from control of epiphyton and nuisance filamentous algae: Implications for shallow lake management. Sci. Total Environ. 705, 135958. doi: 10.1016/j.scitotenv.2019.135958 31838421

[B37] Yang C. Shi X. Nan J. Huang Q. Shen X. Li J. (2022). Morphological responses of the submersed macrophyte Vallisneria natans along an underwater light gradient: A mesocosm experiment reveals the importance of the Secchi depth to water depth ratio. Sci. Total Environ. 808, 152199. doi: 10.1016/j.scitotenv.2021.152199 34890676

[B38] Yu Q. Wang H. Li Y. Shao J. Liang X. Jeppesen E.. (2015). Effects of high nitrogen concentrations on the growth of submersed macrophytes at moderate phosphorus concentrations. Water Res. 83, 385–395. doi: 10.1016/j.watres.2015.06.053 26196308

[B39] Yu Q. Wang H. Wang H. Xu C. Liu M. Ma Y.. (2022). Effects of high ammonium loading on two submersed macrophytes of different growth form based on an 18-month pond experiment. Front. Plant Sci. 13. doi: 10.3389/fpls.2022.939589 PMC9330597 35909745

[B40] Yuan H. Wang H. Zhou Y. Jia B. Yu J. Cai Y.. (2021). Water-level fluctuations regulate the availability and diffusion kinetics process of phosphorus at lake water-sediment interface. Water Res. 200, 117258. doi: 10.1016/j.watres.2021.117258 34058482

[B41] Zhang X. Guo K. Lu C. Awais R. M. Jia Y. Zhong L.. (2020a). Effects of origin and water depth on morphology and reproductive modes of the submersed plant Vallisneria natans. Global Ecol. Conserv. 24, e01330. doi: 10.1016/j.gecco.2020.e01330

[B42] Zhang P. Kuramae A. van Leeuwen C. H. A. Velthuis M. van Donk E. Xu J.. (2020b). Interactive effects of rising temperature and nutrient enrichment on aquatic plant growth, stoichiometry, and palatability. Front. Plant Sci. 11. doi: 10.3389/fpls.2020.00058 PMC7028819 32117394

[B43] Zhang M. Wen S. Wu T. Wang S. Li X. Gong W.. (2022). Patterns of internal nitrogen and phosphorus loadings in a cascade reservoir with a large water level gradient: Effects of reservoir operation and water depth. J. Environ. Manage. 320, 115884. doi: 10.1016/j.jenvman.2022.115884 35940015

[B44] Zhi Y. Liu Y. Li W. Cao Y. (2020). Responses of four submersed macrophytes to freshwater snail density (Radix swinhoei) under clear-water conditions: a mesocosm study. Ecol. Evol. 10, 7644–7653. doi: 10.1002/ece3.6489 32760554 PMC7391322

[B45] Zhou J. Leavitt P. R. Zhang Y. Qin B. (2022). Anthropogenic eutrophication of shallow lakes: is it occasional? Water Res. 221, 118728. doi: 10.1016/j.watres.2022.118728 35717711

